# Treatment of Aneurysmal Bone Cyst with Endoscopic Resection and Bone Allograft with Platelet-Rich Plasma: A Case Report

**DOI:** 10.1055/s-0043-1764459

**Published:** 2023-05-03

**Authors:** Alfred Ferré-Aniorte, Eduard Alentorn-Geli, Xavier Cuscó, David Barastegui, Roberto Seijas, Pedro Álvarez-Díaz, Jordi Navarro, Patricia Laiz, Ramón Cugat

**Affiliations:** 1 Fundación García Cugat, Barcelona, Catalunya, Spain; 2 Instituto Cugat, Hospital Quirónsalud, Barcelona, Spain; 3 Mutualidad de Futbolistas Españoles - Delegación Cataluña, Barcelona, Spain; 4 Universidad Internacional de Cataluña, Barcelona, Spain

**Keywords:** aneurysmal bone cyst, platelet-rich plasma, calcaneus

## Abstract

An aneurysmal bone cyst (ABC) is a rare bone tumor usually observed in long bones. The surgical treatment of this pathology is often related to high recurrence rates, so novel biological techniques can help to enhance tissue regeneration and bone consolidation. We present a case of a patient with ABC of the calcaneus treated with an endoscopic resection followed by grafting with an autologous-based matrix composed of allograft bone chips and autologous platelet-rich plasma (PRP) in semisolid and liquid states. Patient demonstrated excellent defect filling in both magnetic resonance imaging and radiologic exams and returned to pre-injury activity with no recurrence at 2 years follow-up. Endoscopic curettage together with allograft bone and autologous PRP is effective in treating ABC patients and could be a good adjuvant treatment to prevent reinjury and enhance consolidation.

An aneurysmal bone cyst (ABC) is a benign, solitary, and osteolytic bone tumor usually located in the metaphyseal region of long bones. 1 2 Despite its benign nature, it can lead to important sequelae such as pathologic fractures if not diagnosed early. ABC can resolve spontaneously, but can also become aggressive, coursing with bone cortical destruction, which increases its probabilities to become malignant. 2

Surgical treatment is recommended for patients with large cysts in weight-bearing areas due to increased risk of pathologic fracture or persistent pain despite adequate nonoperative strategies. Several procedures have been described to manage ABC, including wide resection, curettage, radiotherapy, sclerotherapy, and embolization. 3 4 5 Wide resection has demonstrated acceptable recurrence rates. 2 However, it is a highly invasive technique that can lead to several comorbidities, 6 so curettage is being the preferred approach, despite having higher recurrence rates.

The application of platelet-rich plasma (PRP) has been hypothesized to enhance tissue repair and regeneration in musculoskeletal injuries. 7 Regarding bone injuries, Sanchez et al reported good results in the application of PRP and bone allograft in patients with bone nonunions, showing that its use is safe and may be effective in osseous pathologies. 8

However, to the best of our knowledge, no studies have evaluated the efficacy of the application of PRP and bone allograft after endoscopic curettage in reducing recurrence rates or helping with bone consolidation.

The purpose of this report was to present a case of a patient diagnosed with ABC of the calcaneus treated with an endoscopic curettage where the cavity was filled with allograft bone chips combined with PRP in semisolid and liquid states.

## Case Presentation



**Video 1** The video shows the process of first endoscopic view, the removal of the neoplastic tissue, and the membrane curettage. Then, the video shows the filling of the cavity with bone chips obtained from allograft femoral heads, together with the matrix of platelet-rich plasma (PRP) and allograft bone. After all this process, it can be observed the final PRP injection directly into the cavity.


A 23-year-old male was referred to our service complaining of a swelling on the lateral aspect of the calcaneus with 2 months of history not related to any traumatic event. He presented no history of previous foot or ankle injury.

**Fig. 1:**
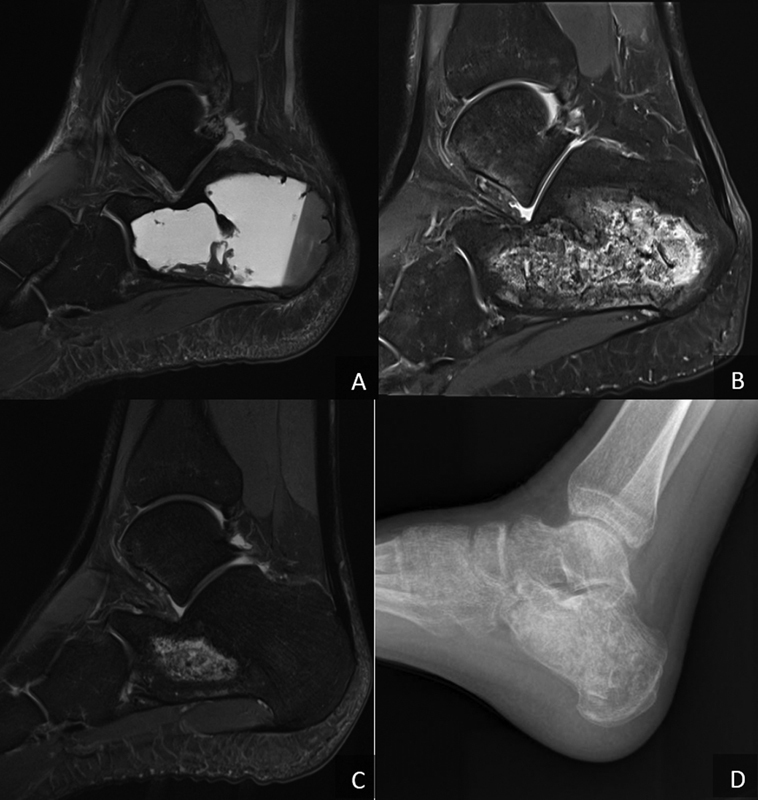
Composition of figures demonstrating magnetic resonance imaging evolution from preoperative ( **A** ) to 8 months postoperative ( **B** ) and 13 months postoperative ( **C** ) and radiographic exam at 6 weeks post-surgery ( **D** ).

The radiographic and magnetic resonance imaging (MRI) exams showed a bone cyst filled with liquid, which deformed the posterolateral aspect of the calcaneus causing trabecular bone destruction but maintaining the cortical bone ( Fig. 1A ).

A tissue biopsy was done to confirm suspicions of ABC through a small 1 cm incision on the lateral aspect of the calcaneus at the most prominent zone. The hematic content was aspirated and sent for histopathology study, which confirmed the ABC diagnosis.

**Fig. 2:**
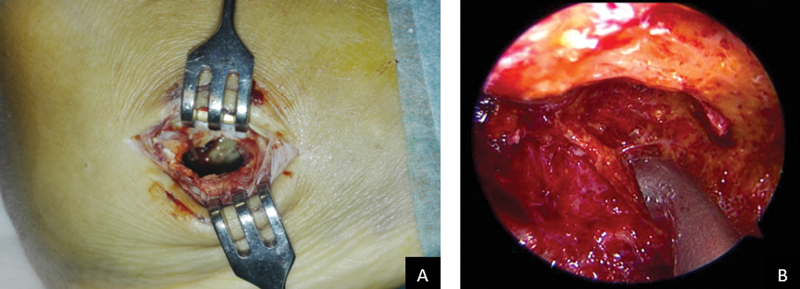
Endoscopic portal and curettage.

**Fig. 3:**
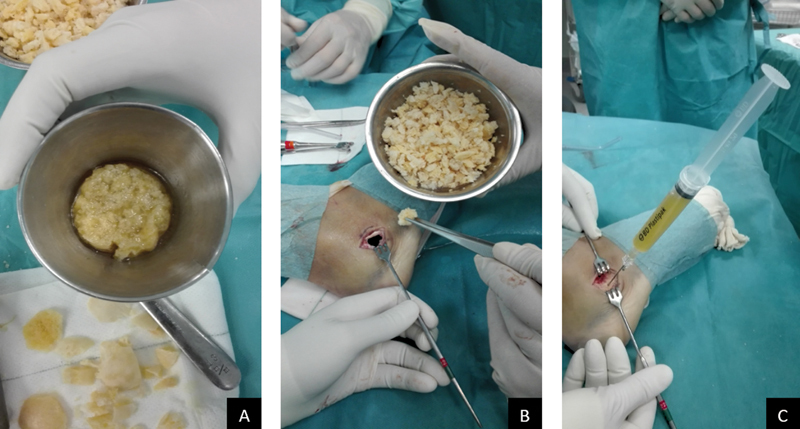
Matrix preparation and platelet-rich plasma (PRP) injection. Clot of allograft bone and PRP ( **A** ); additional allograft administration ( **B** ); final PRP injection ( **C** ).

Endoscopic curettage was performed through the biopsy portal. Tumor membranes were resected until healthy bone walls were observed ( Fig. 2 , Video 1 ). Then, the cavity was filled with bone chips obtained from allograft femoral heads. Those chips were combined with PRP and left for approximately 30 minutes until a semisolid matrix was obtained ( Fig. 3A ). Then, the matrix was placed inside the bone defect together with additional bone graft until bone defect was completely filled ( Fig. 3B ). Additional PRP activated solution (liquid state) was injected ( Fig. 3C ).

PRP preparation was conducted using the Endoret PRGF System (BTI Biotechnology Institute, Álava, Spain). Eighty milliliters of peripheral blood were extracted before surgery and placed in eight 9 mL tubes containing 3.8% of citrate solution. Then, tubes were centrifuged using a BTI System IV (BTI Biotechnology Institute, Álava, Spain) for 8 minutes at 580 g causing the precipitation of red and white cells at the bottom of the tube while PRP stayed at the top. The centrifuged plasma volume was divided by 50%; so, the upper layer and the deeper layer over the precipitated red and white cells were defined as fraction 1 and fraction 2, with lower and higher platelet concentration, respectively.

To prepare the clot, an equal proportion of each fraction was used, resulting in a PRP solution. Then, PRP was activated using 0.02 mL of calcium chloride per milliliter of PRP and maintained at room temperature until the clot was formed.

After surgery, the ankle was immobilized for 3 weeks and recommendation for nonweight-bearing and elevation was provided to the patient. After 3 weeks, the patient showed no pain and a walking boot was recommended for comfort, but still weight-bearing was not allowed. At week 6, consolidation was observed in the radiographic exam at the calcaneus; so, partial weight-bearing was allowed using two crutches ( Fig. 1D ). At 10 weeks, foot orthosis was removed and partial weight bearing was progressed to one crutch. Eight months after surgery, the patient was able to perform activities of daily living and started to run. At 13 months after surgery, patient function improved to pre-injury levels. MRI at 8 and 13 months post-surgery demonstrated good incorporation and consolidation levels of the bone allograft ( Fig. 1 ). At 2 years follow-up, patient reported no complications or reinjury.

Patient consent was obtained and documented in the patients' medical record. This study followed the criteria stated by the Declaration of Helsinki.

## Discussion

ABCs are uncommon benign lesions usually described in individuals at their second decade of life. Several treatments have been proposed for ABC, mostly depending on the location and characteristics of the lesion. 9 10 Godfrey and Gresham stated that small cysts may resolve naturally as results of an intrinsic inactivation by fibrosis and thrombosis of the ABC that would lead to the absorption and recalcification of small cysts and the formation of a cavity filled with thrombotic tissue surrounded by endothelium, fibrous tissue and bone in larger cysts. 11 However, in cases with high risk for pathological fractures or spatial conflicts, surgical treatment is recommended.

High recurrence rate has become a big concern after surgical treatment for ABC. Only wide resection guarantees cure, as other surgical techniques showed a 10 to 30% of recurrence rate. 2 6 Flont et al compared in a retrospective study the clinical outcomes and complications between wide resection technique and curettage. Their results showed a nonsignificant tendency to improve pain, range of motion, and muscle strength in a curettage group. However, follow-up at 9.2 years showed two cases of recurrent ABC in curettage group compared with none on the wide resection group. 12 Aiba et al showed good results after endoscopic curettage procedure in 30 patients. However, they reported a 10% recurrence rate and an average time to bone solid union of 3.2 months. ^3^

Those results confirmed the idea that wide resection is the best option as it represents lower recurrence rates. Still, several postoperative complications have been related to this technique, such as neuropathy, infection, or nerve root injury. 6 Those findings have led practitioners to choose less invasive options such as curettage with or without bone grafting. 1 3 6 12 13 Cyst curettage, although, can lead to incomplete membrane resection due to the presence of multiple cavities with difficult access and suboptimal visualization. Therefore, the use of endoscope to enhance visualization and perform a complete resection is recommended.

There are cases where surgical intervention of ABC is not feasible due to access difficulties to the injury site. For those, alternative treatment options have been described. 9 Selective artery embolization of the feeding arteries can interrupt the blood supply to the injury site, promoting the start of the intrinsic inactivation process. 9 Moreover, the administration of denosumab drug has also shown good results in the treatment of a select patients with ABC despite the low quality of the literature available. Denosumab is a Food and Drug Administration-approved drug for the treatment of giant cell tumor that induces and antiresorptive effect on the osteoclasts inhibiting the cascade that promotes abnormal bone reabsorption. 14

In this report, after cyst removal, the cavity was filled with allograft bone chips combined with PRP to prevent pathologic fractures. Bone defect can be filled with several materials such as cement, animal bone or human bone, both allografts, or autograft. 2

The role of PRP in musculoskeletal injuries has been wildly studied, showing important effects in fibrogenesis, angiogenesis, immunomodulation, and tissue regeneration after its application. 7 When treating bone defects, however, the role of PRP is still not clear. A recent review reported good preclinical results, but their translation into clinical practice was limited due to the low quality of the literature and treatment heterogeneity. 15 When applied together with bone allograft, PRP has showed good results in bone pathologies such as nonunion fractures but, to the best of our knowledge, no research has been done studying the application of PRP and allograft bone in ABC.

We propose the application of an endoscopic curettage together with bone allograft filling combined with adjuvant PRP both as semisolid and liquid states to assure complete cyst removal and enhance bone integration and regeneration.

Only one study evaluated the outcomes after application of autologous PRP in patients with bone cysts. 16 Twelve children diagnosed with both juvenile and ABCs were treated with PRP as a part of a more complex treatment including elastic stable intramedullary nailing and artificial bone substitute. They reported good results, with no refracture and very good functional outcomes at follow-up in all patients.

Because recurrence can occur up to several years after surgery, it might be argued that recurrence after the presented treatment strategy is not discarded. However, this study showed good results when compared with previous research that evaluated other surgical techniques such as wide resection or endoscopic curettage with or without bone graft. 2 This suggests that the performance of this technique could become a good complement to more traditional managements to reduce recurrence rate while maintaining good functional outcomes. In addition, the application of PRP is safe and does not increase the risk of ABC recurrence.

To the best of our knowledge, this is the first report evaluating the use of endoscopic-assisted ABC resection with cavity filling using a combination of bone allograft with PRP. More research must be done to evaluate long-term outcomes and recurrence rates compared with other surgical procedures in larger samples.

## Conclusion

In this report, we propose the use of PRP together with bone allograft and endoscopic curettage in the treatment of ABC of the calcaneus, which could become a good alternative to more conventional treatments in improving recurrence rates and bone regeneration.

## References

[1] Rosenblatt J Koder A Understanding unicameral and aneurysmal bone cysts Pediatr Rev 2019 40 02 51 59 30709971 10.1542/pir.2015-0128

[2] Mascard E Gomez-Brouchet A Lambot K Bone cysts: unicameral and aneurysmal bone cyst Orthop Traumatol Surg Res 2015 101(1, Suppl):S119 S127 25579825 10.1016/j.otsr.2014.06.031

[3] Aiba H Kobayashi M Waguri-Nagaya Y Treatment of aneurysmal bone cysts using endoscopic curettage BMC Musculoskelet Disord 2018 19 01 268 10.1186/s12891-018-2176-6 30053808 PMC6064064

[4] Ibrahim T Howard A W Murnaghan M L Hopyan S Percutaneous curettage and suction for pediatric extremity aneurysmal bone cysts: is it adequate? J Pediatr Orthop 2012 32 08 842 847 23147629 10.1097/BPO.0b013e31825d3619

[5] Aiba H Kobayashi M Waguri-Nagaya Y Treatment of simple bone cysts using endoscopic curettage: a case series analysis J Orthop Surg Res 2018 13 01 168 10.1186/s13018-018-0869-z 29976220 PMC6034211

[6] Elsayad K Kriz J Seegenschmiedt H Radiotherapy for aneurysmal bone cysts: a rare indication Strahlenther Onkol 2017 193 04 332 340 27957589 10.1007/s00066-016-1085-6

[7] Anitua E Nurden P Prado R Nurden A T Padilla S Autologous fibrin scaffolds: when platelet- and plasma-derived biomolecules meet fibrin Biomaterials 2019 192 440 460 30500725 10.1016/j.biomaterials.2018.11.029

[8] Sanchez M Anitua E Cugat R Nonunions treated with autologous preparation rich in growth factors J Orthop Trauma 2009 23 01 52 59 19104304 10.1097/BOT.0b013e31818faded

[9] Chan S-K Muhamad Ariffin M H Aneurysmal bone cyst (ABC) of the C2 vertebra Cureus 2022 14 08 e27735 10.7759/cureus.27735 36106253 PMC9444833

[10] Subramaniam C S Mathias P F Aneurysmal bone cyst J Bone Joint Surg Br 1962 44-B 01 93 101 14039574 10.1302/0301-620X.44B1.93

[11] Godfrey LW, Gresham GA . The natural history of aneurysmal bone cyst Proc R Soc Med 1959 11 900 905 10.1177/003591575905201102 PMC1870818 13850355

[12] Flont P Kolacinska-Flont M Niedzielski K A comparison of cyst wall curettage and en bloc excision in the treatment of aneurysmal bone cysts World J Surg Oncol 2013 11 109 10.1186/1477-7819-11-109 23701661 PMC3669013

[13] Farouk H A Saladin M Senna W A Ebeid W All-endoscopic management of benign bone lesions; a case series of 26 cases with minimum of 2 years follow-up SICOT J 2018 4 50 10.1051/sicotj/2018041 30465648 PMC6250076

[14] Alhumaid I Abu-Zaid A Denosumab therapy in the management of aneurysmal bone cysts: a comprehensive literature review Cureus 2019 11 01 e3989 10.7759/cureus.3989 30972268 PMC6443517

[15] Roffi A Di Matteo B Krishnakumar G S Kon E Filardo G Platelet-rich plasma for the treatment of bone defects: from pre-clinical rational to evidence in the clinical practice. A systematic review Int Orthop 2017 41 02 221 237 27888295 10.1007/s00264-016-3342-9

[16] Rapp M Svoboda D Wessel L M Kaiser M M Elastic Stable Intramedullary Nailing (ESIN), Orthoss® and Gravitational Platelet Separation–System (GPS®): an effective method of treatment for pathologic fractures of bone cysts in children BMC Musculoskelet Disord 2011 12 45 21314981 10.1186/1471-2474-12-45 PMC3046000

